# Self-Pierce Riveting: Development and Assessment for Joining Polymer—Metal Hybrid Structures in Lightweight Automotive Applications

**DOI:** 10.3390/polym15204053

**Published:** 2023-10-11

**Authors:** Ramakrishnan Sankaranarayanan, Navasingh Rajesh Jesudoss Hynes, Maria P. Nikolova, Jolanta B. Królczyk

**Affiliations:** 1 Department of Mechanical Engineering, Mepco Schlenk Engineering College, Sivakasi 626005, India; 2 Faculty of Mechanical Engineering, Opole University of Technology, 76 Proszkowska St., 45-758 Opole, Poland; j.krolczyk@po.edu.pl; 3 Department of Material Science and Technology, University of Ruse “Angel Kanchev”, 8 Studentska Str., 7017 Ruse, Bulgaria; mpnikolova@uni-ruse.bg

**Keywords:** self-pierce riveting, automobiles, polymers, metals, multi-material structures, deformation

## Abstract

In recent years, the transportation industry has faced the challenge of cutting costs, meeting increasingly stringent environmental regulations, and significantly increasing transportation volumes. One approach to meeting these challenges is to develop new, improved transportation vehicles using new materials and innovative joining techniques. Multi-material structures are becoming an alternative to body parts. Self-pierce riveting technology plays a crucial role in this process, and hybrid structures depend exclusively on it. In this article, recent advances in self-pierce riveting technology are analyzed to meet today’s challenges and future multi-material applications.

## 1. Introduction

Continuous evolution can be recognized in automobiles with the incorporation of new features. Also, various materials have been researched and deployed for manufacturing automobiles as part of the evolution. In particular, weight reduction in automobiles without compromising the integrity and function of automobiles is the prime motive behind research into all these materials. The overall weight of the automobile is an important concern for automobile manufacturers, as every exclusive model launched during the last five decades of the 20th century had an excessive weight issue. However, countries in the European Union have addressed the weight issue seriously and formulated stringent laws to tackle exhaust gas emissions, as excess automobile weight leads to high exhaust gas emissions [1,2]. In a revolutionary move, the European Union became the first region to implement environmental regulation of automobiles, providing an example for the rest of the world. Periodic increases in fuel prices are also a factor of concern in reducing automobile weight for the world’s countries. In these contexts, research works have focused on introducing automobiles with novel structures that are lightweight in nature [3,4]. The processing of novel materials and the production of structures are not possible without the effective contribution of processing techniques. Self-pierce riveting (SPR) is one such potential processing method. SPR provides high-speed fastening solutions and supports the application of modern materials and structures with multiple combinations [5].

Much focus has been paid to material science, as structures made out of materials—especially lightweight materials—are suitable for the reduction in fuel consumption and exhaust gas emission. Also, modern materials have to be explored as an effective alternative solution for already existing materials, as these conventional materials have been utilized extensively, and the availability of these materials is a factor of concern in the long run. For example, the introduction and evolution of composite materials have revolutionized the industrial sector, especially the automobile field. Composite-based materials provide solutions with unique combinations of mechanical properties. Furthermore, corrosion resistance and inhibition of chemical reactions are benefits of these multi-constituent composite materials [6,7]. Also, cost-effectiveness, design flexibility, and mechanical reliability make composites the most preferable materials for manufacturing firms [8,9]. As stated above, multi-material structures are in demand, and the presence of conventional–modern material combinations such as metal–metal, metal–composite, and polymer–metal can be witnessed in different applications [10,11]. For example, steam turbines comprising diaphragms fall under the multi-material combination of structures. Multi-material structures provide effective solutions in place of conventional structures in automotive applications and for overcoming the limitations of conventional materials through weight reduction, cost savings, meeting emission laws, energy conservation, etc. [12]. 

**Figure 1:**
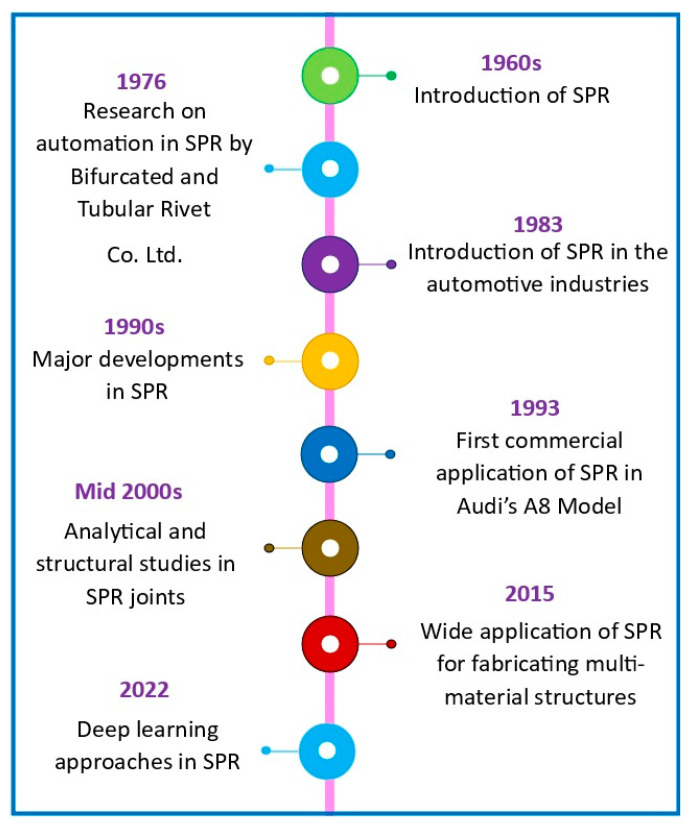
Timeline of significant developments in self-pierce riveting.

Multi-materials can also be witnessed in electromagnetic devices and heavy equipment [13]. Similarly, polymer–polymer combinations of multi-material structures can be accomplished using thermoplastic/thermoset matrix-based composites [14,15]. The combination of metal–polymer structures exhibits excellent mechanical properties where metal contributes to strength and ductility, and polymer material provides physicochemical resistance through weight reduction. However, modern and novel materials cannot be deployed in various applications without suitable processing techniques, for example joining technology. Moreover, the complete replacement of modern materials is not feasible due to specific mechanical property requirements. The combination of modern and conventional materials can meet industrial needs. Exclusive joining techniques have to be adopted to achieve multi-material structures. Moreover, these joining mediums have to be manufacturer-friendly with short cycle time, safety features, ease of execution, etc. [16,17,18]. Adhesive means of bonding and mechanical (riveting/bolting)-based joining are the reliable joining mediums currently available for the realization of multi-material structures [19,20,21,22,23]. The evolution of self-pierce riveting is shown in Figure 1. SPR originated in the 1960s and has experienced significant growth in the last three decades [24,25]. Automation of SPR was attempted and demonstrated by experts from Bifurcated and Tubular Rivet Co., Ltd. [26]. The possibilities of applying SPR in the automotive sector were explored in 1983. Automotive structures made out of aluminum using SPR and strength at the joints were compared with joints made with spot welding [27,28]. Remarkable progress was observed during the 1990s in the context of research studies and applications [29]. Large-scale commercial application of SPR was achieved in 1993 through joint ventures between Audi and Henrob. During the mid-2000s, analytical and structural studies were initiated by researchers of SPR, which added value to this novel riveting process [24]. The application spectrum of SPR increased from the year 2015 onwards, during which structures with dissimilar materials were witnessed [30]. In recent years, deep learning approaches have been experimented with using SPR for the enhanced implementation of the process through joining process optimization [31].

Self-pierce riveting is considered a promising candidate for joining of metal–metal, and polymer–metal hybrid structures in the automotive industry. The SPR technique is envisaged to increase the performance of automobiles by reducing their weight. The efficacious joining technique of self-pierce riveting is reviewed in detail in the current paper.

## 2. Self-Pierce Riveting

### 2.1. Process Overview

The SPR technique was established for making joints in two or more materials through a cold mechanical joining method. Competitive SPR technology can be applied to the joint formation of similar or dissimilar structures [24]. Here, the rivet is pierced from the top side of the material and advanced through the material until anchoring takes place at the bottom portion of the material. The rivet is directed by the appropriate die at the bottom, and the respective die allows the deformation of the rivet to form the anchoring shape. Self-pierce riveting technology started its journey five decades ago but has significantly developed in the last three decades only due to demand for joining aluminum materials in the automotive sector [24,32]. 

The prime differences between these technologies and the classical method of riveting are the elimination of alignment and pre-punching [33]. Four decades previously, a successful attempt was made using the self-pierce riveting technique by Bifurcated and Tubular Rivet Co. Ltd. A waterproof handle was joined to the lid of a can [34], and the SPR technique was easily adopted for the automation [26]. Aluminum usage in the automotive field has been increasingly steadily reducing fuel consumption and toxic exhaust gases, which has resulted in the replacement of mild steel considerably. 

Resistance spot welding made this possible by joining aluminum alloys with the existing materials in the presence of problems. The properties of aluminum, such as high electrical as well as thermal conductivity and a default oxide film on the surface, made the resistance spot-welding process a challenging task, as these properties led to high electrode wear and frequent surface conditioning of the electrode. Furthermore, the aluminum surface must be dealt with carefully due to its sensitivity. Welding aluminum involves higher energy consumption than steel. Apart from that, dissimilar structures cannot be welded by the resistance spot-welding process.

Self-pierce riveting converted those problems into opportunities and emerged as a successful alternative technique. The process is effectively applied in civil construction and automobile fields [35,36], white goods [24], road signs [37], etc. Among them, the automobile sector emerged as a chief area for self-pierce riveting applications [24]. SPR emerged as a promising joining solution for structures made with a combination of aluminum and steel. In comparison to the spot-welding process, SPR is less sensitive to joining parameters. For example, consider the melting points of different alloys [38]. SPR exhibits prominent advantages over the mechanical-clinching technique, especially in joining dissimilar materials by providing efficient interlocking with higher load-carrying strength than mechanical clinching [39]. 

SPR entirely depends on the mechanical means of joint formation rather than fusion for the formation of joints. The science behind the joining makes self-pierce riveting a beneficial method for joining different or similar materials in the absence of surface preparation and thermal degradation. In the automobile industry, self-pierce riveting is being used in combination with adhesives to boost stiffness. It further improves the performance of the automobile by reducing noise, vibration, and harshness.

### 2.2. A Regular Self-Pierce Riveting System

A regular self-pierce riveting system, as shown in the figure, comprises basic parts such as the die and punch. There are subsystems for driving the punch and feeding the rivet. The power and control units are also available for efficient riveting operations. A C-frame consists of a punch and feeding system at the top and die arrangements at the bottom. Process monitoring systems are among the modern additions to existing systems. These sophisticated systems monitor the parameters of the process, such as the thickness of the stack, length of the rivet, punch displacement, and setting force, for the optimum output. 

**Figure 2:**
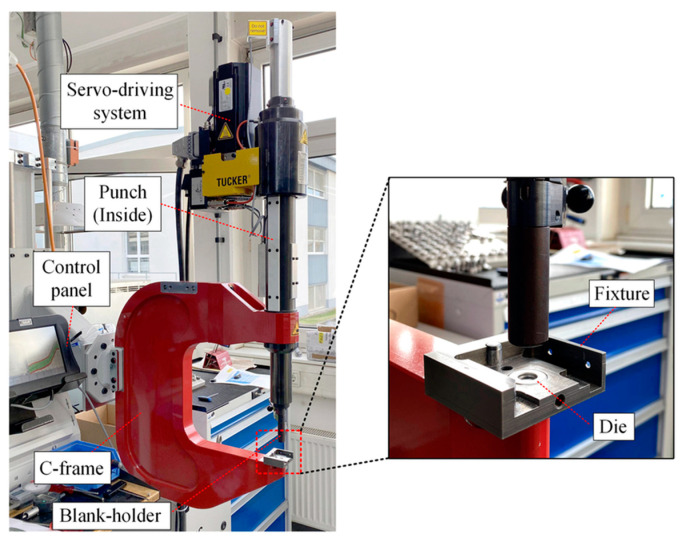
A regular self-pierce riveting system with a servo system by Tucker GmbH. Reprinted with permission from Ref. [43]. Copyright 2022, Elsevier, Zhao et al.

A warning system as a feedback mechanism was also provided for alerting in case of any deviations from the allowable limits [24]. Either servo or hydraulic means of the driven mechanism are employed in the self-pierce riveting system. However, there are a few exceptional cases where gunpowder is used for driving [40]. The servo-driven system is especially renowned for producing structures and components for automobiles due to its lightweight and easy automation. Research was carried out in the context of weight reduction with the help of composites and steels [41]. In the servo-driven system (Figure 2), the position of the rivet from which it is driven classifies the process as a pushing or punching process. The pushing process is executed in such a way that the rivet is pierced through the workpiece by the punch, at which point the progressive force is applied, and the application of force continues until the target position is achieved. In contrast, in the punching process, the rivet receives an impact punch with a specific speed to reach the target position. These two methods of self-riveting were compared through research, and it was concluded that the punching process was more effective than the pushing process due to localized distortions and inconsistencies in the pushing process [40,42,43]. 

Further research established that gunpowder-driven impact self-pierce riveting also experienced fewer localized distortions during the execution of the riveting process than with hydraulic-powered ones [40].

### 2.3. Principles behind the Operation

**Figure 3:**
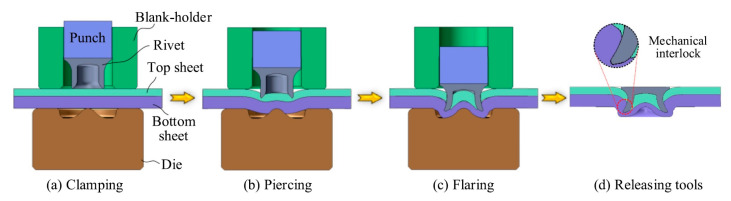
Major specific stages of self-pierce riveting. Reprinted with permission from Ref. [43]. Copyright 2022, Elsevier, Zhao et al.

Four major, specific stages of self-pierce riveting are shown in Figure 3. The first stage of self-pierce riveting is the clamping stage, where the bottom flat surface of the blank holder is placed over the workpiece against the placement of the die beneath the workpiece by the application of force. The bottom and top holding with a certain amount of applied force provides the perfect clamping, as shown in Figure 3a. The optimum clamping force is decided based on the joint stacks. Clamp force at the lower side enables material flow in the bottom region for minimizing the localized work-hardening and avoiding cracks. The clamping force at the higher side facilitates localized work-hardening and provides sufficient squeezing in the joint region [24]. The material flow at the bottom sheet is facilitated by the low clamping force, resulting in reduced local work hardening. On the other hand, a high clamping force provides sufficient crushing to the adhesive at the joining region and escalates the local work hardening [24,43]. The second stage of self-pierce riveting is called the piercing stage, where the workpiece is penetrated by the rivet through the application of pushing or punching force on the rivet by the punch. Flaring of the rivet is very minimal at this stage, but considerable penetration takes place, as shown in Figure 3b. The piercing stage depends on the material to be riveted, as soft and hard materials behave differently [43]. The workpiece made of soft material experiences penetration at the top sheet, and the hard material experiences early rivet flaring. Meanwhile, the hardness of the rivet also influences this stage, as the softer the rivet material, the more easily flaring occurs. Thus, the rivet and workpiece material combination is a very important factor to be considered for easy penetration with minimal flaring. 

Flaring takes place at the pre-final stage of self-pierce riveting, where the rivet is given a punch or push against the workpiece. Anchoring is accomplished via this flaring phenomenon by mechanical means, and the entire sheet is interlocked. Flaring is made possible by employing workpiece resistance and die constraint. The hardness of the rivet material, rivet setting velocity, and geometry of the die play a significant role in flaring [11,24]. Gap generation may occur between sheet materials due to the dissimilarities in the behavior of deformation. But these gaps are reduced once the force is applied via the rivet head. A predetermined force decides the end of punching, and this stage reaches its endpoint [24]. The fourth and final stage of self-pierce riveting is releasing. Retrieval of the nose piece and punch occurs, and they are returned to their working positions for the next cycle of riveting. Die design is especially important, as the design influences all four stages of SPR.

#### 2.3.1. Deformation Behavior of the Workpiece and Rivet Material

**Figure 4:**
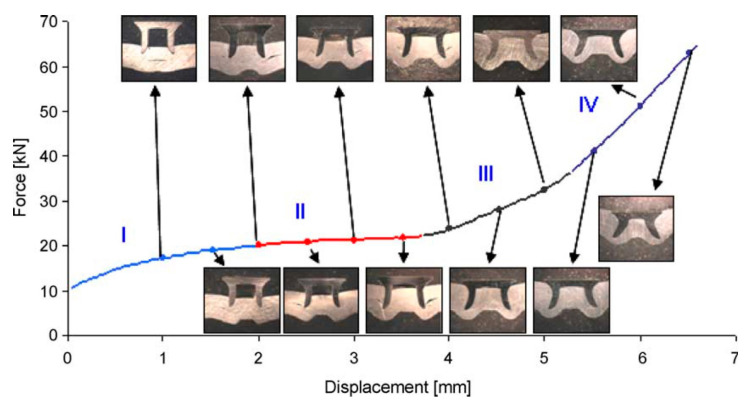
Representation of the influence of force on displacement during the self-pierce riveting process Reprinted with permission from Ref. [44]. Copyright 2005, Elsevier, Cai et al.

The deformation behavior of the workpiece and rivet material in the self-pierce riveting process makes this process another mode of categorization. Experiments were carried out by Cai et al. using two AA5754 aluminum sheets of 1.2 mm gauge. Figure 4 represents the influence of force and displacement during the self-pierce riveting process of a total thickness of 2.4 mm of AA5754 aluminum sheets [44]. The typical behavior is shown in Figure 4 as a representation of the influence of the force on displacement during the self-pierce riveting process [44]. These four categories were based on the crucial events in a riveting process. The first event (I) involves the local bending of the sheets and penetration of the rivet into the top sheet. Further penetration of the rivet continues until the rivet reaches the lowest sheet during the second event (II), and the die receives more material flow in its volume. Expansion of the rivet occurs in the third event (III), where the entire closing from top to bottom sheet is accomplished. The final event (IV) ends with a mechanical means of interlocking by the placement of the rivet head at the respective position [45,46].

#### 2.3.2. Findings of Previous Research

**Figure 5:**
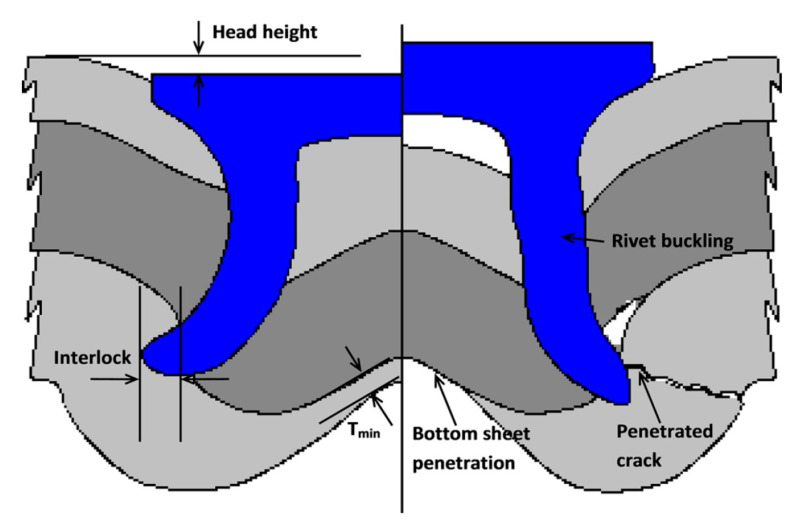
Quality of a joint in self-pierce riveting process including flaws. Reprinted with permission from Ref. [24]. Copyright 2017, Li et al.

Further understanding of the force versus displacement behavior was carried out, and those findings revealed that the rivet and die geometry, the thickness of the sheet, and the nature of the material could influence the flow of the curve [44,47]. A force versus displacement behavior curve was used for the validation of the simulation [48], which was carried out with sheets of dissimilar thickness and hardness of the rivet [49]. The curve indicates that considerable forces are needed in the third and fourth events as the rivet experiences a higher level of resistance for deformation as well as penetration. It behaves differently depending on the different parameter settings of the rivet and the stacking of the workpiece. The same curve can be applied for process monitoring. Other factors, such as the length and hardness of the rivet, the workpiece material’s strength and thickness, the geometry of the die, the order of the stacking, and the number of workpiece materials, also influence the flow of the force versus displacement curve. The criteria for the quality of joints will vary from application to application. However, three critical criteria are common, irrespective of the applications, for achieving excellent joints. The height of the rivet head, the residual bottom thickness of the workpiece (T_min_) as represented in Figure 5, and the interlocking span are remarkable criteria to be considered. The use of die-sided sheets requires a large deformation of the rivet head protrusion, which leads to an increase in stress and, as a result, damage to the rivet head. A residual bottom thickness of the aluminum alloy workpiece of up to 1.0 mm will not cause rivet head deformation. The number and degree of filling of the ring grooves influence the load-bearing capacity of the rivet without head deformation. Under a cross-tensile load, only a low strength of 600 N could be achieved. High load-bearing capacities under shear and cross-tensile loads up to 4 kN could be reached using the rivet with head deformation [24].

The elevation of the rivet head is the key deciding factor for its appearance. The other influences on the height of the rivet head are the joint consolidation, the span between the top sheet and rivet head, damage caused by the rivet on the topmost sheet, the strength of the joint, etc. 

The interlocking span is the most influential factor in the quality of the joint. The interlocking span is the measure of the strength of the mechanical locking between the bottommost sheet and the rivet. At the same time, the residual bottom thickness of the workpiece after riveting is not a vital factor for the strength of the joint. But this is the key factor for corrosion, noise, vibration, and harshness (NVH). Manufacturers of automobiles anchor the rivet head on the inner side of the structure and place the joint buttons on the underside of the automobile’s wet areas so that a minimum residual bottom thickness will be maintained that avoids the breakage of the rivet and, understandably, corrosion resistance. A few more factors are also under consideration, such as joint button cracks, rivet buckling, the span between the topmost sheet and rivet head, and the gaps within the layers of the sheet. The self-pierce riveting process excellently suits the unification of both similar and dissimilar materials. Cast, wrought, and extruded aluminum alloys can be riveted for joint formation. Grades of such wrought aluminum alloys include 6xxx, 5xxx, etc. Similarly, high-strength, advanced high-strength, and mild steels are also possible materials for SPR [24]. The general requirement for self-pierce riveting for workpiece materials is sufficient ductility, specifically for the lowest layer material that is close to the die. This may avoid stern cracks in the region of the joint button. 

**Figure 6:**
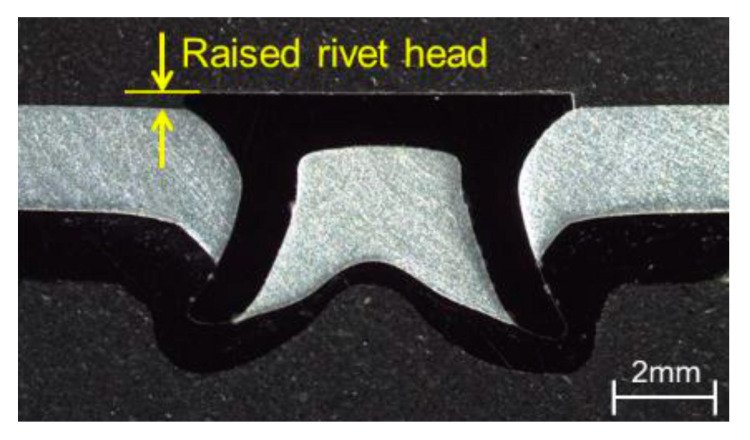
High-quality self-pierce rivet joints produced by placing the thicker aluminum sheet on the top side and the thinner steel sheet at the bottom side. Reprinted with permission from Ref. [51]. Copyright 2018, Elsevier, Ma et al.

The other notable necessity is the possession of sufficient hardness as well as strength for the rivet material. This property only facilitates the penetration of the rivet into the material to be riveted with sufficient anchoring in the absence of buckling or squeezing. The placement of the workpiece material is to be reordered based on its characteristics. For example, the placement should be either topmost or middle in the case of the brittle nature of the material. If there is an unavoidable situation, the placement on the bottom side should be carried out with thermal assistance. The ability of the riveting could be influenced by the differences in the thickness of the top and bottom layer materials when two-layer stacking is employed. It is advisable to use the thicker one at the bottom and thinner one at the top. In certain conditions, the thicker material is placed on top due to the difficulties in accessibility and other concerns. Here, the selection of the die and rivet materials is important, along with their designs, to ensure effective anchoring quality. These kinds of rivetability studies were executed on various materials. One such study revealed that aluminum alloy and steel should be placed at the top and bottom, respectively, in the steel-aluminum alloy combination of materials to achieve excellent rivetability. At the same time, the bottom sheet that is next to the die should be thicker than that of the topmost sheet [50]. Nonetheless, the same order of placement need not be mandatory, as specified above, since the sophisticated self-pierce process made successful joints of high quality by placing the thicker aluminum sheet on the top side, as exhibited in Figure 6.

**Figure 7:**
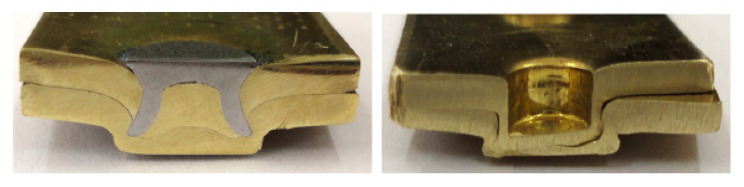
Self-pierce riveting (left side), clinched riveting (right side): cross-sections. Reprinted with permission from Ref. [54]. Copyright 2015, Elsevier, Xing et al.

Meanwhile, the method of keeping the steel at the top and aluminum alloy at the bottom provides excellent joints compared to the reverse method [50]. The feasibility of applying self-pierce riveting has been studied by several researchers. The major constraint of the magnesium alloys for involving them with the self-pierce method is its low ductility, especially at ambient temperature. It results from the hexagonal lattice arrangements within its structure. But the ductility behaves differently, exhibiting an increasing trend as the temperature increases. This was evident in the case of induction-heated magnesium alloy AZ31 at 280 °C joined as either a top or bottom material with self-pierce riveting and clinching [52]. The applications of self-pierce riveting are not limited to steel, magnesium, and aluminum. It can be extended to other materials such as titanium and copper. The great ductility of copper makes it easy to apply in the SPR process, and it can be joined with aluminum [53]. Similarly, the copper–copper combination of self-pierce riveted joints is also possible [54]. The same combinations were joined through self-pierce riveting and clinching processes as shown in Figure 7. Even though titanium is difficult to deform, it can be riveted by this process when its temperature is elevated above 700 °C [55,56]. 

**Figure 8:**
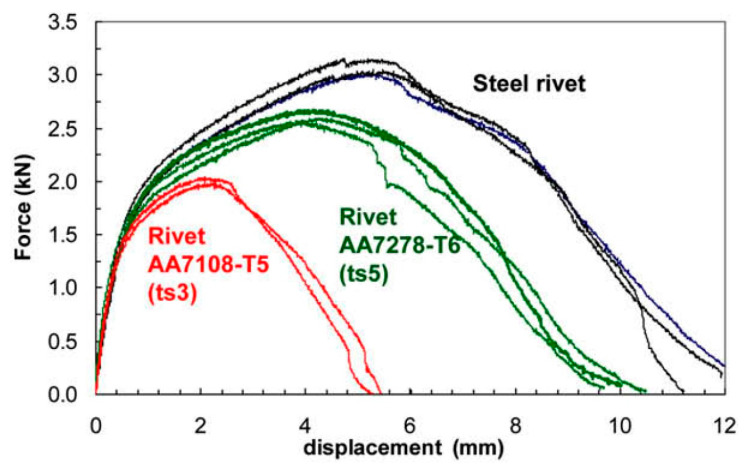
Correlation of the steel rivet with aluminum rivets with self-pierce riveting technology Reprinted with permission from Ref. [58]. Copyright 2010, Elsevier, Hoang et al.

Sandwich structures can also be produced with the assistance of self-pierce riveting. Sandwich structures are typically composite structures with at least two different combinations of materials with different properties, characteristics, thickness, etc. These combinations take the materials to the next level of performance. For example, the sandwich structure is made with steel–polymer–steel–aluminum alloy combinations with different thicknesses using the SPR method [57]. Furthermore, the steel–polymer combination of sandwich structure is also feasible via this novel riveting process [24]. The possibility of placing the aluminum rivets in place of steel rivets in the classical die setup was explored by joining two pieces of AA6060 sheets of 2 mm thickness with AA7278/T6 rivets. Figure 8 displays the moderate static strengths despite the low interlocking [58].

## 3. Factors and Response Analysis Related to Self-Pierce Riveting

### 3.1. Implications of the Quantity of Pre-Straining

**Figure 9:**
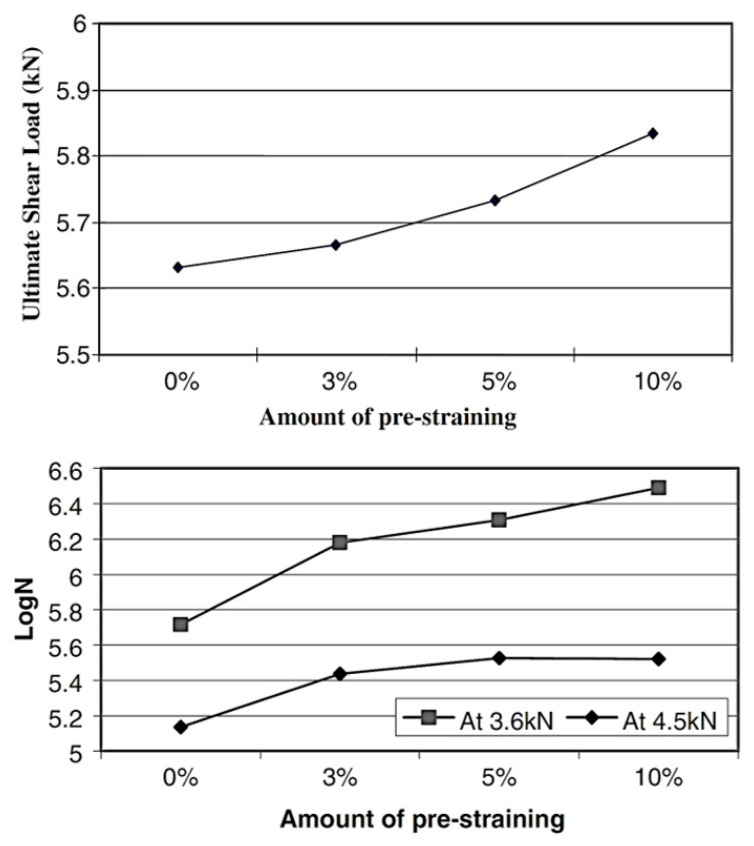
Implications of the quantity of pre-straining. Reprinted with permission from Ref. [59]. Copyright 2006, Elsevier, Han et al.

The joint strength of self-pierce riveting is greatly impacted by the category of material. In addition to this, the treatment of the material, like work hardening, influences the strength [59]. For example, the stamping process, which strains and hardens the respective material being processed, is highly influential in the manufacturing of automobiles. The impact of stamping was analyzed by investigating the pre-straining (Figure 9) and its influences with the aid of joints made via self-pierce riveting of aluminum alloy. Likewise, age hardening enhanced the material strength, which was reflected in the self-pierce riveted joints. The outcomes clearly revealed that the more pre-straining, the greater the ultimate shear strength [59].

### 3.2. Implications of the Edge Distance

**Figure 10:**
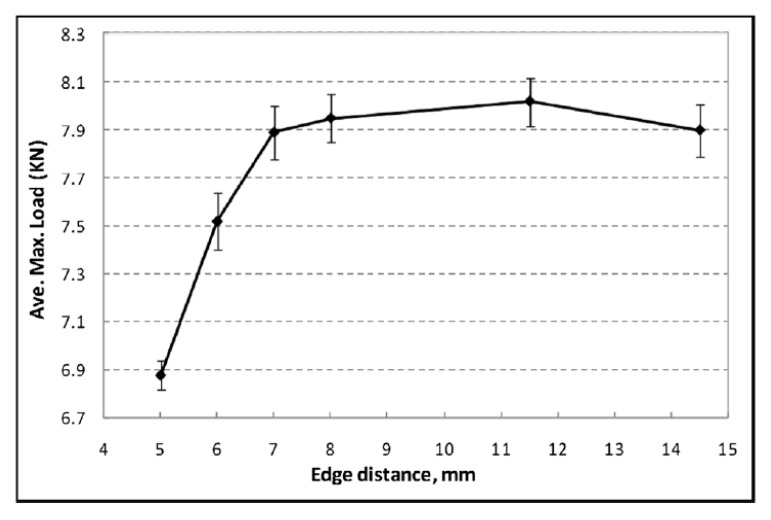
Implications of the edge distance on the strength concerning lap shear load. Reprinted with permission from Ref. [60]. Copyright 2012, Elsevier, Li et al.

**Figure 11:**
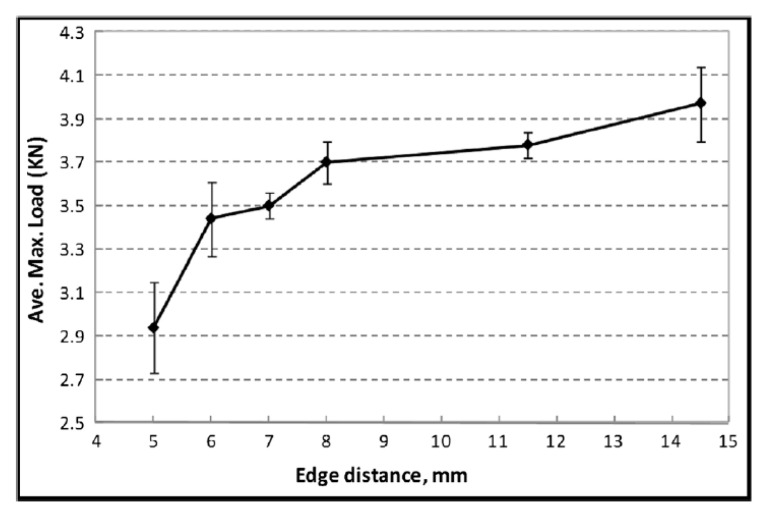
Implications of the edge distance on the strength concerning T-peel load. Reprinted with permission from Ref. [60]. Copyright 2012, Elsevier, Li et al.

The implications of the edge distance on the lap as well as T-peel strength were investigated. Figure 10 and Figure 11 represent the influences, respectively [60]. These graphs exhibit that the higher the edge distance, the higher the strength for both lap and T-peel loads. Similar results were found by Sunday for different workpiece widths [27].

### 3.3. Displacement Behavior of the Various Self-Pierce Riveted Joints

**Figure 12:**
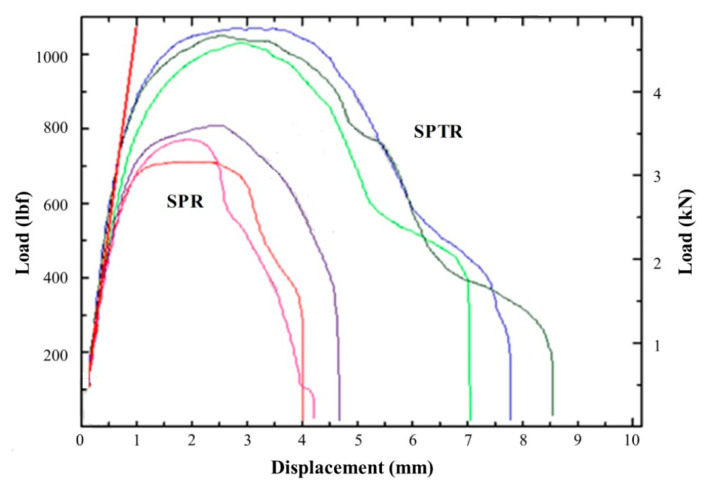
Displacement behavior of the various self-pierce riveted joints. Reprinted with permission from Ref. [32]. Copyright 2020, Elsevier, Rao et al.

**Figure 13:**
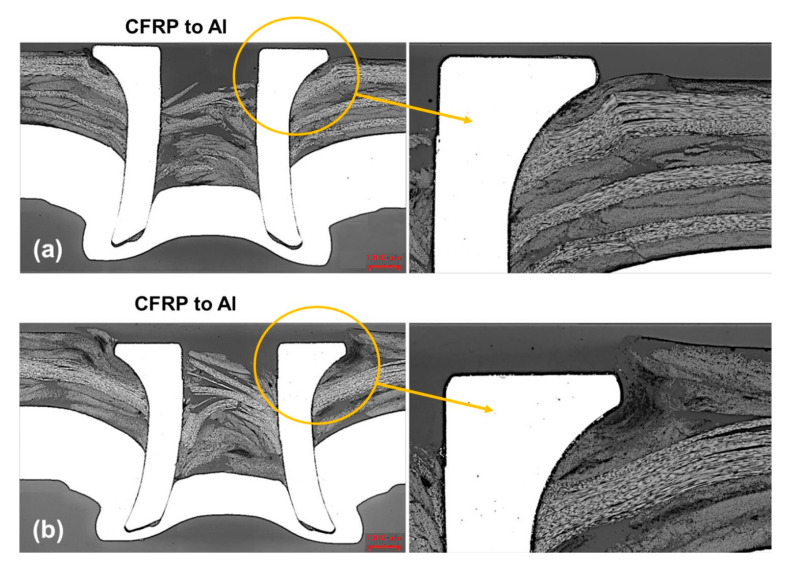
Representation of cross-sectioned SPR joints with CFRP–AA6111, (**a**) flush-rivet head and (**b**) without flush-rivet head. Reprinted with permission from Ref. [61]. Copyright 2018, Elsevier, Rao et al.

A comprehensive study of the displacement behavior of the various self-pierce riveted joints with the application of loads was conducted for the Nylon 6 (PA6) matrix Al joint combination, and results were obtained in terms of ductility as shown in Figure 12. In addition to that, the behavior of the joints was good. Nevertheless, aluminum (Al)/glass-fiber-reinforced polymer (GFRP) and glass-fiber-reinforced polymer (GFRP)/aluminum (Al) combinations also possessed remarkable ductility but later exhibited higher strength, whereas the aluminum (Al)/carbon-fiber-reinforced polymer (CFRP) combination showed marginal ductility. The strength was somewhat lower in comparison with the Nylon 6 (PA6) matrix and aluminum joints. The displacement behavior of the carbon-fiber-reinforced (CFR) PA6 composite is shown in Figure 12. SPR joints with CFRP–aluminum alloy (AA6111) combinations are exhibited in Figure 13.

### 3.4. Failure Behavior of Various Self-Pierce Riveted Joints

The self-pierce riveting process and nature of the reinforcement of fibers were analyzed to determine the extent of their influence on the accomplishment of joint strength [24]. This was achieved by analyzing the failures that occurred. Two distinct failures were recognized. One type of failure occurred when a rivet was pulled out with the least amount of damage due to the bearing. The second occurred due to bearing damage through the rivet “pull-in” on the upper side. The latter failure can be termed a bearing failure. 

**Figure 14:**
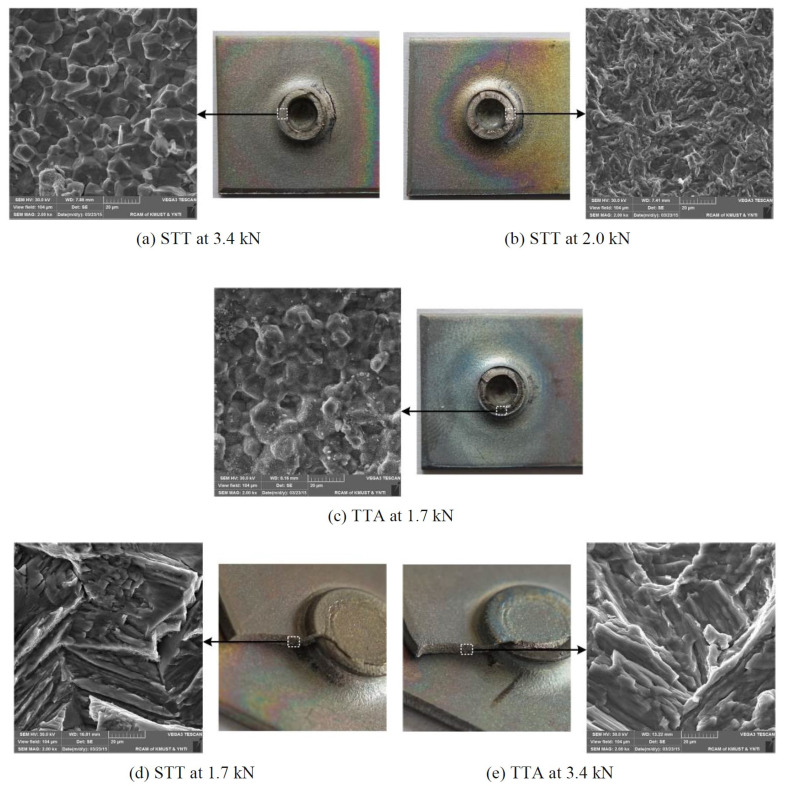
Fatigue fracture analysis over self-pierce riveted joints at macro and micro level. Reprinted with permission from Ref. [55]. Copyright 2016, Elsevier, Zhang et al.

“Pull-out” was the repeated mode of failure during the placement of the Al alloy on the top side. Consequently, all of these cases possessed closer strength values. This placement of the Al alloy on the upper side provides better anchoring of the rivet on the Al alloy. The bearing phenomenon was absent in the PA6 matrix during the separation of Al/PA6 blanks as a consequence of low strength and good ductility. The same phenomenon was experienced in GFRP and CFRP workpieces. Fatigue fracture analysis was carried out over similar and different combinations of joints made using the self-pierce riveting technique at the micro and macro levels [55]. Through this approach, the fatigue behavior was determined, and the respective analysis is displayed in Figure 14. 

The riveted joint was created with two sheets of titanium alloys (Figure 14a). TA1 (GR1) titanium alloy is annealed at 540 °C. It possesses excellent corrosion resistance, high weldability, good impact toughness, and excellent room-temperature ductility. It has 240 MPa tensile strength, 122 Vickers hardness, and a 100 GPa modulus of elasticity. The riveting failed at 3.4 kN, and the macro-level examination revealed that the failure occurred in the rivet region. However, the exact occurrence was found via micro-level examinations, where cracks due to fatigue appeared in the upper sheet’s penetrated hole region. The appearance of the surface indicates the crystalline nature, which in turn exhibits brittleness. Nonetheless, the nature of the failure was different for the same combination at the 2 kN fatigue failure level (Figure 14b). The nature of fatigue failure was ductile in the form of stripes as far as appearance was concerned. Similar results can be seen in Figure 14c, in line with Figure 14a, where the only difference was the additional annealing process for relieving the stresses in the latter case with a 1.7 kN fatigue load. The phenomenon of fatigue failure resulted in cracks starting from the shank region of the rivet and lasting until the breakage of the lower sheet due to the damage as a result of interlocking. The generation of fractures was brittle in nature, with stripes. The fatigue load (1.7 kN) was the lowest amongst the riveted joints in the absence of annealing for the joint, as shown in Figure 14d. In comparison to all joints, the surface appearance of the joints with annealing (Figure 14c,e) was smoother than that of the joints made without annealing (Figure 14a,b,d). However, the annealing process made grain boundaries extremely wide. Moreover, the commencement and journey of the cracks followed a low-energy contour.

## 4. The Prominent Factors Which Influence the Quality of the Joint

**Figure 15:**
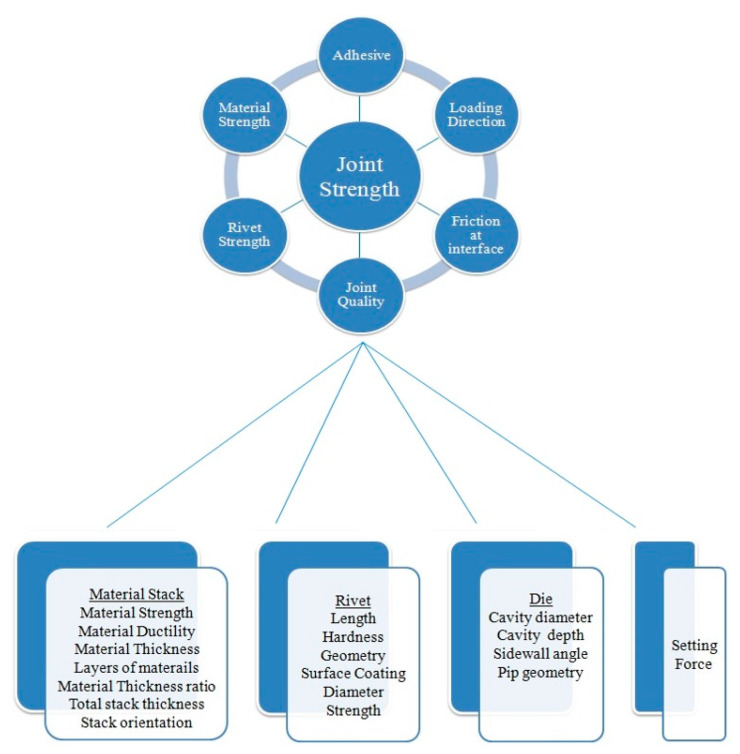
The prominent factors which influence the quality of the joint as well as its strength. Contents of images are adapted with permission from Ref. [24]. Copyright 2017, Li et al.

The design of the rivet and the dies (tools) used in the technique is essential. The rivet should have exact geometry to allow for proper penetration, and the dies should be created to support the materials during riveting. The diameter and length of the rivet are crucial to the success of the process. They should be selected based on the thickness and type of materials being bonded. The hardness of the rivet material should match that of the components being attached. If the rivet is too soft, it may distort significantly during the piercing operation. The die material should be chosen to withstand the forces involved in the SPR process. Furthermore, the hardness of the die material should be appropriate for the materials being used. The riveting machine’s pressure should be carefully managed. Excessive pressure can cause material deformation or damage, whereas insufficient pressure might result in an incomplete junction. The dwell time is the amount of time that the pressure is sustained after the rivet has entered the material. This parameter is critical for allowing the joint to form properly. The prominent factors that influence the quality of the joint as well as its strength are represented in Figure 15. Despite several factors and conditions, the self-pierce riveting process is suitable for making hybrid structures, for example, the combinations of metal and polymer, steel and composite, as well as aluminum and steel. In the combination of metal composites, it is advised to use the composite material at the top layer as it is brittle in nature. The top placement provides sufficient time for heat inducement on the brittle material, which aids the deformability of the brittle material and results in improved interlocking [24]. In certain areas, this type of heating is insufficient, and an external heat source is employed for heat assistance. The application of this riveting technology can be extended and taken to the next level by applying it to different new materials (very-high-strength steels, press-hardened steels, casting materials, etc.) and new combinations of materials (fiber-reinforced composites). Further exploration of the mechanical behavior of these joints is needed, and the same is true for the modeling of the SPR process. Comprehending the residual stress generated during the self-pierce riveting process is also vital for future improvements.

The successful progress in SPR technology can be encouraged by extending its boundaries of applications to more materials, such as high-strength steels, forged hardened steels, castings, and composite materials with reinforcement. Apart from this, a deeper analysis of the mechanical performance of the successfully applied materials can be conducted for further enhancements. 

Software-based modeling analysis is also widely needed in this type of riveting. The order of placement of materials in multi-material riveting is also a matter of concern, as this orientation affects the joining [24]. The joint stiffness significantly affects the static and fatigue performance of the self-pierce rivet joints [53]. The prime failure mode for these riveted joints is rivet pull-out, especially in static conditions where the separation of the lower sheet occurs from both the rivet and the upper sheet. The term “relative slip” is closely associated with joining technology, and the same association prevails here with the SPR process, determined by the fatigue load-carrying capacity. A “relative slip” value is directly proportional to the fatigue load. Moreover, the facts on crack initiation as well as propagation can be inferred from this value, so that failures can be predicted well in advance [54]. Similarly, research findings show that “relief annealing” also influences the fatigue performance of these joints but has less impact on static strength [55]. There is less possibility of uniform failure modes for the different self-pierce rivet joints due to the involvement of multi-materials or similar materials of different properties in the riveting process [56].

The exclusiveness of the rivet–die combination for particular riveting restricts usage, which is one of the limitations of this process. The thickness of the bottom sheet greatly influences the rivet’s flaring and, thus, the mechanical interlocking. The greater the thickness, the better the flaring and interlocking of the rivet. It is advisable to apply SPR technology where the cost of the riveting can be justified by the mechanical performance of the joint, as the cost involved is greater for executing this type of riveting [61]. More understanding of the influence of residual stress on riveted joint performance is required. However, modeling and performance analysis are more difficult for the self-pierce riveting process due to the nature of its complexity [24]. All these challenges and limitations provide a clear road map for researchers to explore innovations.

## 5. Scope of Self-Pierce Riveting among Other Contemporary Processes

The scope, capacity, or breadth of a process can be determined by comparison to other existing joining methods or techniques in the same field of applications. Here, aluminum alloys are mostly applied in the automobile sector. So, the joints made by different joining methods with aluminum alloys were compared here. The mechanical strength of self-pierce riveting was compared with resistance spot welding. The materials used were aluminum alloys for the joining [27,62]. 

The results revealed that self-pierce riveted joints possessed either higher or lower lap shear strength than spot-welded joints in the case of dissimilar stacks of the material. But tensile strength was lower for self-pierce riveting as compared to resistance spot welding. In the case of fatigue strength, self-pierce riveting exceeded the performance of resistance spot welding, and fatigue results matched the results [29]. Similar inferences were made for aluminum alloy AA6111 joints [63] and for AlMg3W19 joints [64]. Among self-pierce riveting, resistance spot welding, and clinching processes, for shear and static peel strength, self-pierce riveting showed the highest strength and resistance spot welding and clinching the least. 

**Figure 16:**
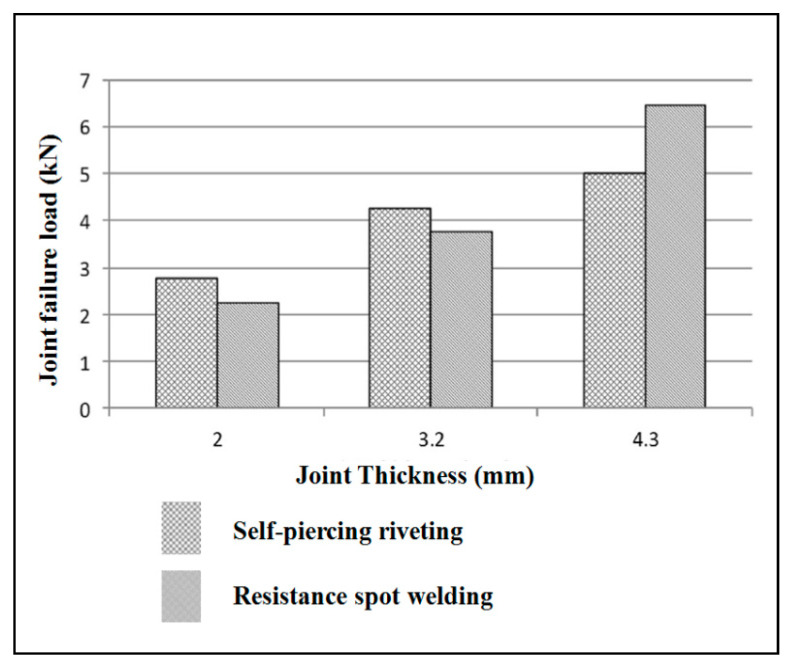
The behavior of the static shear strength of aluminum alloy joints made by self-pierce riveting and resistance spot welding. Reprinted with permission from Ref. [24]. Copyright 2017, Li et al.

**Figure 17:**
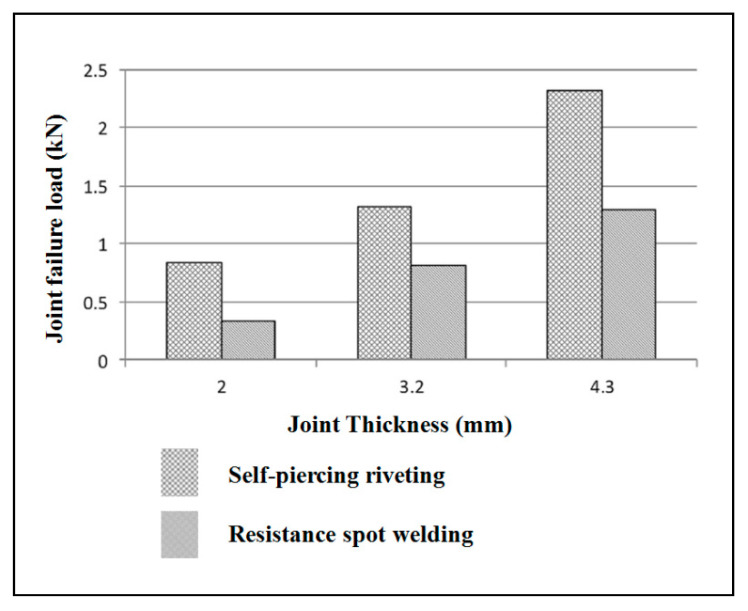
The behavior of the static peel strength of aluminum alloy joints made by self-pierce riveting and resistance spot welding. Reprinted with permission from Ref. [24]. Copyright 2017, Li et al.

The other property called the static strength of the joint, was compared for self-pierce riveting and resistance spot welding [65]. Al5052 aluminum alloy with a dissimilar stack thickness was used to make the joints. It is annealed at 345 °C. Al5052 is extremely resistant to corrosion, and it possesses a high magnesium content, making it the highest-strength non-heat-treatable alloy available. It has good finishing characteristics and excellent workability. It has 228 MPa tensile strength, 68 Vickers hardness, and 70.3 GPa modulus of elasticity. In this comparison study, self-pierce rivet joints behaved differently for different thicknesses. For joint thicknesses of 2 and 3.2 mm, lap shear strength was better for self-pierce riveted joints than spot-welded joints. But lap shear strength was low at 4.2 mm thickness. In general, the peel strength was better for self-pierce rivet joints than resistance spot weldments, as presented in Figure 16 and Figure 17 for singular riveting.

An aluminum alloy of AA5754 was also considered for comparison and studying the mechanical strength [66]. Self-pierce riveting responded both positively and negatively in comparison with resistance spot welding. As far as static lap shear strength is concerned, joints made with spot welding showed better results than self-pierce riveting. However, lap shear fatigue strength was inferior for the self-pierce rivet joints. Similar results were obtained, where self-pierce riveting showed better and lower performance on fatigue and shear strength, respectively, compared to resistance spot welding [67]. 

The static strength comparison was carried out for resistance spot welding, spot friction welding, and self-pierce riveting, where T-peel and lap shear strengths were very much alike or even superior in the self-pierce riveting process compared to the other two [62]. Both self-pierce riveting and resistance spot-welding processes were dependent on process parameters and material stack regarding the strength of SPR joints [68]. Based on material stacking or process parameters, higher or lower static strengths were obtained for self-pierce riveting than resistance spot welding. 

For joint thicknesses of 2 and 3.2 mm, lap shear strength was better for self-pierce riveted joints than spot-welded joints. But lap shear strength was low at 4.2 mm thickness. In general, the peel strength was better for self-pierce rivet joints than for resistance spot weldments, as presented in Figure 16 and Figure 17 for singular riveting.

In general, T-peel strengths were high in self-pierce riveting, and cross-tension strengths were high in resistance spot welding [68]. Aluminum alloys with a combination of steel joints were also investigated using self-pierce riveting and resistance spot-welding techniques [69]. In this investigation, the steels were riveted with both the presence and absence of zinc coating. Here, the static strength performance of resistance spot welding was higher than self-pierce riveting. However, the fatigue strength performance of self-pierce riveting was higher than resistance spot welding. A similar comparison showed that the higher the steel strength, the better the fatigue strength for self-pierce joints, but this was not so for spot-welded joints [70]. 

Research on resistance spot welding with high-strength steels revealed that the strength of the substrate influenced joints insignificantly [71]. It was not observed in the self-pierce riveting technology [70]. Significant influences were apparent from the strength of the substrate and the strength of the joints, as the increment in the strength of the substrate reflected the increment in the strength of the joints. The joining of high-strength steels achieved better results with self-pierce joints than spot weldments [72]. Joints made with self-pierce riveting and resistance spot welding using different high-strength steels excelled in both fatigue performance of peel strength, and shear strength of laps were superior in self-pierce joints compared to spot-welded joints at different levels of high-strength steel [73].

Different mechanical joining techniques can be carried out with different materials, such as aluminum alloys, steels, and sandwich (aluminum–polypropylene–aluminum) materials. Similar joints can be made with this material using self-pierce riveting as well. The joints made by the abovementioned methods showed that the strength of the joint and the type of joining process employed was hugely dependent on materials [74]. Research ensured that the fatigue performance of self-pierce riveting was superior to spot weldments [75]. Considering the entire comparison as mentioned above, strength under static conditions was better for self-pierce rivet joints than the joints made with equivalent resistance spot weld joints [24]. The joints made with self-pierce riveting exhibited the same superiority over the resistance spot-welded joints as far as fatigue performance was concerned. 

Analyses were conducted on mechanical clinching, self-pierce riveting, and resistance spot welding and led to comparisons between the above three methods of joining materials [76]. Self-pierce riveting possessed joints with the best fatigue and static strength results among the other two techniques. The reasons for the best fatigue performance were the stress release via the joint interface due to mild slip and the increment in the strength of the material due to work hardening [76]. Other techniques were also compared with self-pierce riveting.

Comparisons were made between the behavior of the performance and strength of the joints by conducting tests on T-peel and lap shear strength. The outcomes of the testing revealed that the self-pierce riveting process is well ahead of spot friction joining techniques and has excellent strength in the joint region. Research has revealed similar results [77]. Apart from the static strength, the absorption of energy is higher for self-pierce joints than spot friction-riveted joints. The T-peel performance of pop rivet joints was studied and compared with the joints made by self-pierce riveting, and pop riveting functioned well in terms of static strength, whereas self-pierce rivets functioned well concerning fatigue strength [77]. The long journey of self-pierce riveting has been covered in several studies. The constant success of this process can be ensured by the appropriate selection of rivet and die materials. However, material selection alone cannot meet the requirements. There are other inputs to be considered carefully, such as the force settings, the geometry of the die, the method of stacking, and the rivet’s tip geometry. Rivets with larger diameters exhibited better strength than the smaller ones [24]. If the top and bottom stacks are made with similar materials, then the increment in the strength of the joints is directly proportional to the increment in either stack or sheet material thickness.

In the case of inequality in stacking thickness, the strength of the joints is dependent on the thickness of the thinner stack materials, and good joint quality would be achieved if a thick bottom stack is used. In addition, the configuration of the rivet tips may influence the strength of the joints. The different geometry of the rivet tip affects the resulting strength of the joints. The nature of the material and the features of the joints determine force settings in the SPR process. Generally, the bottom sheet that is next to the die should be thicker than the topmost sheet. But the same order of placement is not mandatory. Successful joints of high quality were also made by placing the thicker aluminum sheet on the top side, as shown in Figure 6. Commercial self-pierce rivets are usually round, flat, and countersunk, and their nominal diameters are 3.2, 3.9, 4.8, and 5.3 mm, with body lengths of 3 to 9.5 mm. However, limited works are available that deal with the direct influence of these factors on the quality and integrity of joints. 

## 6. Conclusions

Self-pierce riveting is a promising joining process used particularly in automotive and aerospace applications. Though it is a versatile and effective method for joining materials, self-pierce riveting indeed has some limitations. Only ductile materials, typically aluminum and steel, are suitable. It may not be suitable for joining materials that are too hard or brittle. It is effective for joining thin to medium-thickness materials, and it also works effectively for lap joints, although it may not be suitable for other joint configurations. 

The ultimate shear load increases when the percentage of pre-straining increases. When the amount of pre-straining is 10%, the ultimate shear load is 5.84 kN. After an edge distance of 8 mm, the lap shear load remains around 8 kN with a marginal variation. In the case of T-peel load, it drastically increases up to an edge distance of 8 mm, and afterward, there is only a gradual increase. 

The length of commercially available self-pierce rivets is in the range of 3.5 to 14 mm, and they are effective for materials not exceeding 10 mm. These limitations are challenges to be overcome in the future. The design of specific equipment and innovative rivet designs can have a significant impact on the range of material thicknesses that can be effectively joined. 

According to a new study, global sales of self-pierce riveting (SPR) technology will grow at a cumulative annual rate of 26 percent in the next decade, and manufacturers worldwide will consume 45 billion fasteners every year. The promising qualities of the self-pierce riveting technique make it an impressive, quick, and economical joining technique for hybrid polymer–metal multi-structural applications. Automobile industries would immensely benefit from employing this technique. 

Although the automobile industry is the real origin of SPR development, recently, manufacturers of stepladders, air cargo containers, garage doorframes, air conditioners, refrigerators, office furniture, etc., have embraced SPR and expressed interest in this joining technology.

## Data Availability

The data presented in this study are available on request from the corresponding author.
